# Identification of 30 transition fibre proteins in *Trypanosoma brucei* reveals a complex and dynamic structure

**DOI:** 10.1242/jcs.261692

**Published:** 2024-06-03

**Authors:** Manu Ahmed, Richard Wheeler, Jiří Týč, Shahaan Shafiq, Jack Sunter, Sue Vaughan

**Affiliations:** ^1^ Department of Biological and Medical Sciences, Oxford Brookes University, Gipsy Lane, Oxford OX3 0BP, UK; ^2^ Peter Medawar Building for Pathogen Research, University of Oxford, Oxford OX1 3SY, UK; ^3^ Biology Centre CAS, Institute of Parasitology, Branišovská 1160/31, 370 05 České Budějovice, Czech Republic

**Keywords:** Transition fibres, Distal appendages, Ciliogenesis, *Trypanosoma*, Cilia, Flagella

## Abstract

Transition fibres and distal appendages surround the distal end of mature basal bodies and are essential for ciliogenesis, but only a few of the proteins involved have been identified and functionally characterised. Here, through genome-wide analysis, we have identified 30 transition fibre proteins (TFPs) and mapped their arrangement in the flagellated eukaryote *Trypanosoma brucei*. We discovered that TFPs are recruited to the mature basal body before and after basal body duplication, with differential expression of five TFPs observed at the assembling new flagellum compared to the existing fixed-length old flagellum. RNAi-mediated depletion of 17 TFPs revealed six TFPs that are necessary for ciliogenesis and a further three TFPs that are necessary for normal flagellum length. We identified nine TFPs that had a detectable orthologue in at least one basal body-forming eukaryotic organism outside of the kinetoplastid parasites. Our work has tripled the number of known transition fibre components, demonstrating that transition fibres are complex and dynamic in their composition throughout the cell cycle, which relates to their essential roles in ciliogenesis and flagellum length regulation.

## Teaser

**Summary:** We identify and characterise 30 transition fibre proteins in *Trypanosoma brucei*, showing that transition fibres are complex and dynamic structures with roles in flagellum assembly and length regulation.

## INTRODUCTION

Cilia and flagella are highly conserved microtubule-based organelles that project from the cell surface and play essential roles in signalling and/or motility in a range of eukaryotic organisms (Moran et al., 2014). They are assembled from microtubule-based basal bodies, which are composed of nine radially arranged microtubule triplets. Basal bodies and centrioles often exist as a connected pair and duplicate in a cell cycle-dependent manner, with each daughter cell inheriting an older (mature) basal body and a younger (immature) basal body. Only a mature basal body can assemble a cilium or flagellum and contain additional appendages called transition fibres, which are a set of nine blade-like structures that radiate out from the triplet microtubules at the distal end of the mature basal body.

In mammalian cells, assembly of a cilium occurs in G0 phase, when the cell exits the replicative cell cycle. During ciliogenesis, the mature basal body docks to the plasma membrane via distal appendages, which anchors the cilium as it extends out from the cell (Anderson, 1972; O'Hara, 1970). To date, at least ten proteins have been identified at transition fibres in mammalian cells – including C2CD3, CEP83, CEP90 (also known as PIBF1), OFD1, CEP89, SCLT1, CEP164, ANKRD26, CEP19 and FBF1 – and these proteins have important functions in membrane docking and modulation of ciliogenesis (Bowler et al., 2019; Kurtulmus et al., 2018; Tanos et al., 2013; Yang et al., 2018; Kumar et al., 2021). Defective cilia are linked to a class of human diseases known as ciliopathies. Mutations in transition fibre proteins (TFPs) are linked to a number of syndromic ciliopathies, demonstrating the importance of distal appendages for cilium formation and function (Failler et al., 2014; Chaki et al., 2012).

**Fig. 1.**
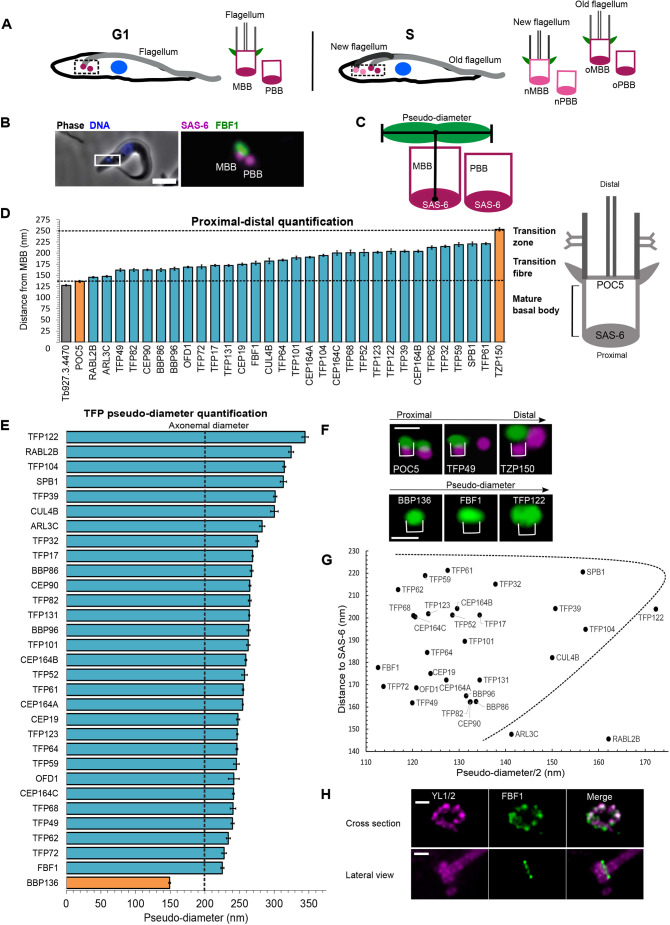
**Spatial organisation of candidate TFPs.** (A) Cartoon representing trypanosome cells; for each cell, the dashed box marks the basal body region depicted to the right, with basal bodies, transition fibres (green) and axoneme (grey) shown. A G1-phase cell is represented on the left, with a single flagellum (light grey) and basal body pair (magenta). An S-phase cell is represented on the right, with a new flagellum (dark grey) and old flagellum (light grey) each with a corresponding basal body pair at the new (pink) and old (magenta) flagellum. Nuclei are shown in blue. MBB, mature basal body; PBB, probasal body; o, old; n, new. (B) Left: merged fluorescence and phase-contrast image of a cell with endogenous protein tagging of FBF1 with mNG (green) and of SAS-6 with mScarlet (magenta). DNA was stained using Hoechst 33342 (blue). Box indicates the basal body region shown in the magnified image (5× zoom) on the right. Right: FBF1 is only located on the MBB and not on the PBB. Scale bar: 5 μm. (C) Cartoon illustration of distance and pseudo-diameter measurements carried out in D and E. See Materials and Methods for more details. (D) Range of measurements of the proximal-distal localisation of candidate TFPs, ordered by their distance from SAS-6. POC5 was used as a control marker for the MBB distal end (as depicted in the diagram on the right). Candidates with a shorter distance from SAS-6 were excluded (grey). TZP150 was used as a control marker for the transition zone. Dashed lines mark mean values for the control proteins. (E) Measurements of TFP pseudo-diameter. Basal body control (BBP136) is shown in orange, and the theoretical axonemal diameter is indicated by the dashed line at 200 nm. Data in D and E are presented as mean±s.e.m. See Table S1 for *n* cells measured. (F) Examples of TFP localisations based on their proximal–distal location (top) and pseudo-diameter (bottom), as in D and E. Top: the indicated mNG-tagged protein is shown in green, SAS-6 is shown in magenta. Bottom: the indicated mNG-tagged protein is shown in green. Brackets indicate the areas included in measurements. Scale bars: 500 nm. (G) Graph showing the approximate location of each TFP group based on pseudo-diameter and distance from the basal body (SAS-6). Data re-plotted from D and E. Dashed line marks the approximate location of the transition fibre. (H) Expansion microscopy of TY-tagged FBF1 (green) colocalised with anti-tubulin antibody staining (YL1/2, magenta). Images are representative of three technical repeats using the same cell line. Scale bars: 500 nm.

Trypanosomes are an excellent system to study ciliogenesis as they possess a basal body pair and a single flagellum that undergo cell cycle duplication, maturation and anchoring in a similar manner to mammalian basal bodies. The flagellum also exhibits the same canonical features as the mammalian cilium, with a transition zone, transition fibres and 9+2 axoneme that undergo cell cycle-regulated assembly (Sherwin and Gull, 1989; Wheeler et al., 2019; Hodges et al., 2010). The genome of trypanosomes encodes many known conserved centriole and cilia genes, including orthologues of many genes involved in human ciliopathies (Broadhead et al., 2006; Hodges et al., 2010; Barker et al., 2014). Therefore, insights gained from trypanosome basal bodies and flagella are likely to apply to other eukaryotic organisms. The main advantages of this model organism for flagellum biogenesis studies are the ability to follow the assembly and duplication of basal bodies, and the ability to unequivocally identify old flagella and assembling new flagella in dividing cells. A G1-phase trypanosome cell has one basal body pair comprising a mature basal body and a probasal body located at the proximal end of a single flagellum (Fig. 1A, left). This flagellum does not disassemble, and a new flagellum grows alongside the existing flagellum in a precise position in preparation for division (Fig. 1A, right). Assembly of the new flagellum begins with basal body maturation, acquisition of transition fibres and docking of the newly mature basal body to the flagellar pocket, which occurs around the time of G1-S transition. New probasal bodies form during S phase next to each mature basal body, and the new flagellum continues growth throughout the rest of the cell cycle (Fig. 1A, right) (Wheeler et al., 2019; Sherwin and Gull, 1989; Gluenz et al., 2011).

Scanning transmission electron tomography studies have confirmed the presence of the canonical nine blades of transition fibres docked to the flagellar pocket in trypanosomes (Trépout et al., 2018). In addition, two widely conserved proteins – CEP164C (CEP164 in mammals) and retinitis pigmentosa-2 (RP-2, also known as XRP2 in mammals) – have been determined to be transition fibre components (Stephan et al., 2007; Harmer et al., 2017; Atkins et al., 2021), and a kinetoplastid-specific protein, TFK1, has been found to localise to transition fibres with CEP164C and RP-2 (Ramanantsalama et al., 2022). In overall molecular terms, transition fibres have broadly been considered a simple structure comprising only a handful of proteins. We leveraged a *Trypanosoma brucei* genome-wide protein localisation resource (Billington et al., 2023) to identify 30 TFPs, which we then characterised by assaying the time of incorporation and position within the basal body region for each protein. Furthermore, we used RNAi-mediated knockdown to assess whether the TFPs identified are important for ciliogenesis. We show extensive protein composition and temporal complexity in transition fibres, and comprehensively map the evolutionarily conserved components and lineage-specific transition fibre complexity that are essential for assembly of a eukaryotic cilium or flagellum.

## RESULTS

### *T. brucei* transition fibres contain at least 30 proteins

A previous genome-wide protein localisation study identified ∼307 putative basal body and basal body-proximal proteins based on localisation to one or two small foci at the proximal end of the single *T. brucei* flagellum (Billington et al., 2023; TrypTag.org). Since transition fibres are present on the mature basal body and surround the barrel of the basal body, proteins that were identified in the previous study as having a clear focus that exhibits a wide lateral signal at the proximal end of a flagellum were taken forward for further investigation here. The transition zone is distal to transition fibres (Fig. 1D, right), and it is possible that our initial screen included proteins with transition zone localisation; however, transition zone proteins are annotated as a separate category in the Tryptag database, and this category was not included in our search criteria. Proximity of the TFP candidates to the mature basal body was further examined by tagging the putative TFPs with an mNeonGreen (mNG) tag at the endogenous locus in a cell line expressing the proximal basal body marker SAS-6 endogenously tagged with mScarlet (Nakazawa et al., 2007; Fong et al., 2014). In total, 31 candidates were taken forward based on the presence of a wide lateral focus of localisation at the mature basal body relative to the tight single focus of SAS-6. An example is the orthologue of the characterised mammalian TFP FBF1, which was found to be located on the mature basal body, as expected (Fig. 1B) (Tanos et al., 2013). All candidate proteins without a detectable characterised orthologue or previous experimental characterisation in *T. brucei* were named TFP, for transition fibre protein, followed by their predicted molecular mass.

Quantification of the distance from SAS-6 and the lateral width of the signal (called pseudo-diameter) was carried out for the mNG signal from each tagged TFP candidate using an automated FIJI script, and measurements of detergent-extracted methanol-fixed cytoskeletons were plotted for each putative TFP (Fig. 1C–E; Table S1 for measurements and number of cells per TFP). Measurements were made on live cells for TFPs where mNG fluorescence signal was lost following detergent treatment (ARL3C, RABL2B and TFP122). Firstly, all candidates that were distal to both SAS-6 and POC5 (Fig. 1D,F) (POC5 is a known distal-end basal body protein but not a TFP; Azimzadeh et al., 2009) but more proximal than the transition zone protein TZP150 (Dean et al., 2016) (Fig. 1D,F) were taken forward. These criteria removed one candidate (Fig. 1D; Tb927.3.4470), bringing the total to 30 proteins. Secondly, all candidates with a pseudo-diameter greater than that of BBP136 (a bona fide mature basal body protein; Dang et al., 2017) (Fig. 1E,F) were taken forward for further study (Fig. 1E; for examples, see Fig. 1F). This criterion did not remove any further candidates. Known TFP orthologues in trypanosomes identified by this method – CEP164A, CEP164B, CEP164C (Hodges et al., 2010) and OFD-1 (Andre et al., 2014) – were found to all fit within the expected region, demonstrating that this method can successfully identify TFPs (Fig. 1D,E). These two measurements resulted in a total of 30 TFPs being taken forward for further study (Fig. S1). Fig. 1G shows a plot of the data from Fig. 1D,E for each of the 30 TFPs based on distance and pseudo-diameter measurements.

To further confirm that the candidates were located at transition fibres, a cell line was generated expressing each TFP candidate endogenously tagged with mNG and the well characterised TFP CEP164A endogenously tagged with mScarlet. For those proteins where we were unable to successfully generate this cell line, we instead assessed colocalisation of the mNG-tagged TFPs with RP-2, which localises to the transition fibres in trypanosomes (Stephan et al., 2007; Atkins et al., 2021), by using the antibody YL1/2 (Fig. S2).

There was a range of pseudo-diameter measurements consistent with the mNG tag on the TFPs being located at various positions along the length of each transition fibre blade (Fig. 1E). TFP122, RABL2B, TFP104, SPB1, TFP39 and CUL4B exhibited the widest pseudo-diameters (300–345 nm) (Fig. 1E,G), and the rest of the TFPs had narrower pseudo-diameters (225–283 nm) (Fig. 1E,G). To compare to the ultrastructural dimensions of transition fibres to our light microscopy measurements, we analysed the distance between the outermost regions of transition fibres by using longitudinal thin-section transmission electron microscopy (Fig. S3). Transition fibres had a mean diameter of 354.3±10.8 nm (mean±s.e.m.; *n*=20), which is a similar range to that of the wider pseudo-diameters in our light microscopy datasets (Fig. S3). Finally, expansion microscopy (Gorilak et al., 2021; Kalichava and Ochsenreiter, 2021) was also carried out on the TFP FBF1 fused to a TY epitope tag at the endogenous locus. This demonstrated a transition fibre arrangement with approximate ninefold symmetry around the microtubules of the basal body, providing further confirmation of these proteins being associated with the transition fibre area of trypanosome cells (Fig. 1H). In summary, this screen identified 30 TFPs in trypanosomes, significantly extending our molecular map of transition fibre components (Table S1).

### Evolutionary conservation of trypanosome TFPs

**Fig. 2.**
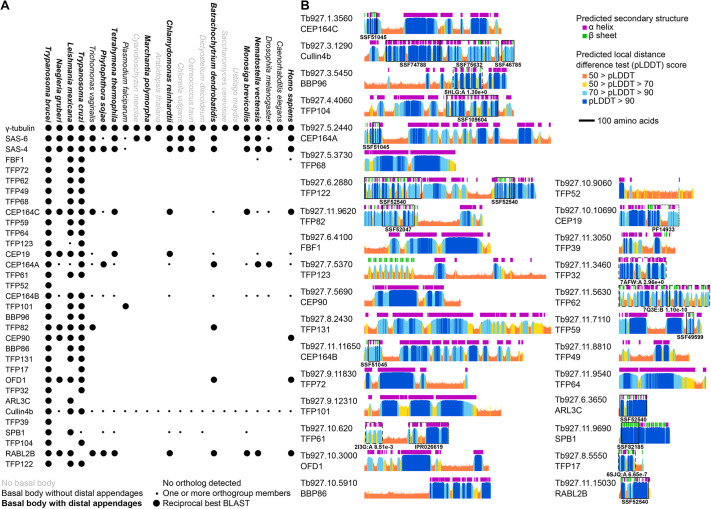
**Evolutionary conservation profile and protein structure prediction of *T.*** ***brucei*** **TFPs.** (A) Presence or absence of orthologues of *T. brucei* TFPs across diverse eukaryotes, in comparison to control tubulin nucleation (γ-tubulin) and basal body (SAS-6, SAS-4) proteins. Large circles indicate presence of an orthologue detectable by reciprocal best BLAST, small circles indicate one or more orthogroup members but not a reciprocal best BLAST. Species fonts indicate their ability to form basal bodies and distal appendages, as previously described (Carvalho-Santos et al., 2011). Cullin4b, CUL4B. (B) AlphaFold2 structure predictions of all *T. brucei* TFPs. As a large majority of predictions showed extended α-helices or no predicted structure, showing the structures is often not highly informative. Instead, we summarise the structures as per-residue plots of predicted local distance difference test (pLDDT) and predicted secondary structure, using the dictionary of secondary structure program (DSSP) on the predicted structure. Protein domains predicted by sequence similarity are marked with boxes: solid boxes for superfamily domains (accessions starting SS), dotted boxes for other database predictions (reserved for when no superfamily domain hit was in that protein region; accessions starting IPR, InterPro; accessions starting PF, Pfam). Dashed boxes show globular regions where we carried out structural similarity comparisons to experimentally determined structures using FoldSeek (PDB IDs are shown alongside E-values).

To analyse the evolutionary conservation of the trypanosome proteins identified in this screen, reciprocal best BLAST and Orthofinder (Emms and Kelly, 2015) were used to identify orthologues in organisms with a known ability to assemble basal bodies (Carvalho-Santos et al., 2011). Our data showed a correlation between the ability to form basal bodies and the presence of orthologues of *T. brucei* TFP-coding genes within the genome, indicating a likely specific requirement in ciliogenesis (Fig. 2A), further confirming the role of these proteins in basal body functions. Nine TFPs identified in our screen had a detectable orthologue in at least one basal body-forming eukaryotic organism outside of kinetoplastid parasites (RABL2B, CEP164 proteins, CEP19, OFD1, CEP90, FBF1, TFP101, TFP82 and SPB1), with the most widely conserved being the CEP164 proteins, CEP19 and RABL2B. The remaining trypanosome TFPs were only detected in other kinetoplastids. However, this bioinformatics analysis only used the trypanosome amino acid sequence. There is a large evolutionary distance between some organisms, so known orthologues in these organisms might not appear in this analysis. From this analysis we can conclude that, although transition fibres appear to display many structural and functional similarities across ciliated cells, trypanosome TFPs are either not well conserved across the wide range of ciliated cells in eukaryotic organisms or do not have a well conserved primary sequence. In this set we included four organisms (*Caenorhabditis elegans*, *Drosophila melanogaster*, *Plasmodium falciparum* and *Trichomonas vaginalis*) that have basal bodies but, when studied using electron microscopy, do not appear to have structures resembling transition fibres (Carvalho-Santos et al., 2011) (Fig. 2A). However, both *T. vaginalis* and *D. melanogaster* have orthologues of the CEP164 proteins, and recently, CEP164, FBF1 and CEP89 have been experimentally characterised in *D. melanogaster*, revealing localisation to the distal end of the basal bodies, which suggests additional functions for these proteins (Hou et al., 2023).

Fewer than half of the *T. brucei* TFPs have predicted protein domains detectable on the basis of sequence (as determined by querying the superfamily database; Wilson et al., 2009), and these predicted domains rarely span a large portion of the protein sequence. Therefore, we explored the AlphaFold2 predicted structures of the TFPs (Jumper et al., 2021), using lineage-optimised input multiple sequence alignment (Wheeler, 2021), to gain insight into potential functions (Fig. 2B). We found that many of the TFPs are predicted to be extremely α-helix rich, with FBF1, TFP68, TFP64, TFP101 and TFP131 predicted to be essentially entirely α-helical. The predicted structures for most TFPs have long helical regions, sometimes also with small globular domains, such as the N-terminal WW domain (SSF51045) of the CEP164 family, and long C-terminal α-helix domains. These have the potential to be physically large; with a pitch of 5.4 Å and 3.6 amino acids per turn, 1000 amino acids could form a 150 nm α-helical arm. For protein regions with a predicted globular structure but no corresponding superfamily domain, we searched for the protein with the most similar experimentally determined structure in Protein Data Bank (PDB) using FoldSeek (van Kempen et al., 2024). TFP17 was found to have a predicted structure similar to that of *T. brucei* BILBO1 (E-value of 6.65×10^−7^ versus PDB 6SJQ) (Florimond et al., 2015), a major component of the flagellar pocket, which the trypanosome basal bodies dock onto for ciliogenesis. We found that TFP62 has a predicted structure similar to that of mouse inturned (INTU; E-value of 1.10×10^−10^ versus PDB 7Q3E, chain B), which is part of the ciliogenesis-associated CPLANE complex (Langousis et al., 2022). Only one protein had any similarity to the predicted structure of TFP32 (E-value 2.96×10^0^ versus PDB 7AFW, chain A). With such a marginal E-value, this should likely be discounted; however, it is notable that this hit is β-catenin (CTNNB1), which has known cilia-associated functions. The C-terminal region of TFP122 has a domain with predicted structure similar to that of a pleckstrin homology domain, which are typically involved in phosphatidylinositol binding. Consistent with its wide pseudo-diameter, TFP122 might therefore be involved in membrane interaction. From these data we can conclude that although there appears to be a set of TFPs at least conserved between humans and trypanosomes, the nature of TFPs means that they might diverge in sequence too greatly to identify orthologues in distant species across eukaryotes by sequence methods alone.

### Specific TFPs are recruited before and after basal body duplication

**Fig. 3.**
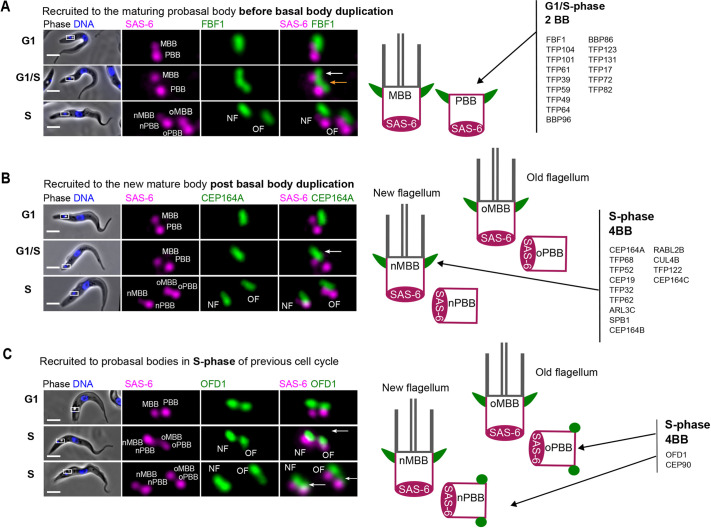
**TFP recruitment before and after basal body duplication.** (A) Example of a TFP recruited before basal body duplication. Left: images of a live cell expressing endogenously tagged FBF1 (mNG, green) and SAS-6 (mScarlet, magenta) at G1 phase, G1-S transition (G1/S) and S phase. At G1/S, a focus of FBF1 can be observed on the existing mature basal body (white arrow) and a second focus of FBF1 can be observed on the maturing body prior to basal body duplication (orange arrow). Right: cartoon representing localisation of the listed TFPs, which are recruited before basal body duplication in trypanosomes (basal bodies in magenta, transition fibres in green and axonemes in grey). (B) Example of a TFP recruited after basal body duplication. Left: images of a live cell expressing endogenously tagged CEP164A (mNG, green) and SAS-6 (mScarlet, magenta) from G1 phase to S phase. At G1/S, CEP164A can only be observed on the existing mature basal body (white arrow), and a second focus of CEP164A is not observed on the maturing body prior to basal body duplication (marked by the appearance of four SAS-6 foci). Right: cartoon representing localization of the listed TFPs, which are recruited after basal body duplication. (C) Example of a TFP recruited to probasal bodies in S phase of the previous cell cycle. Left: images of a live cell expressing endogenously tagged OFD1 (mNG, green) and SAS-6 (mScarlet, magenta) from G1 phase to S phase. After basal body duplication, OFD1 foci can be observed on the mature basal bodies (white arrow, middle row) and subsequently observed arriving on both probasal bodies (white arrows, bottom row). Right: cartoon representing localization of the listed TFPs, which are recruited in the S phase of the previous cell cycle. In A–C, DNA was stained using Hoechst 33342 (blue). Boxes in the merged phase-contrast and fluorescence images indicate the basal body region, which is shown at higher magnification in the fluorescence images on the right (5× zoom). Images are representative of *n*=100 cells for each cell line. The phenotypes shown in A and B were observed for all cells. In C, a third of cells in early S phase had signal on probasal bodies. Scale bars: 5 μm. BB, basal body; MBB, mature basal body; NF, new flagellum; nMBB, new mature basal body; nPBB, new probasal body; OF, old flagellum; oMBB, old mature basal body; oPBB, old probasal body; PBB, probasal body.

The acquisition of transition fibres by a mature basal body is a critical step in docking and ciliogenesis (Wei et al., 2015; Tanos et al., 2013; Schmidt et al., 2012). Docking of a new mature basal body and growth of a new flagellum narrowly precedes the formation of two new probasal bodies in trypanosomes (Fig. 1A) (Gluenz et al., 2011; Wheeler et al., 2019). This feature allowed us to discover whether TFPs were recruited to new the mature basal body before or after basal body duplication. SAS-6 is one of the earliest proteins to arrive at the start of new probasal body assembly (Hu et al., 2015). Therefore, we used cell lines expressing SAS-6 tagged with mScarlet with each mNG-tagged TFP as a temporal marker of probasal body formation (Fig. 3A–C). Endogenously tagged FBF1 localised to a single wide structure overlying the mature basal body in cells with two SAS-6 foci (Fig. 3A, G1-phase cells). In a proportion of cells with two SAS-6 foci there was a V-shaped signal representing the transition fibres of two separate but closely positioned mature basal bodies (Fig. 3A, cells at G1-S transition). This demonstrates early recruitment to the new mature basal body before basal body duplication. In total, 15 TFPs were recruited to the new mature basal body prior to ciliogenesis and represent early markers of transition fibre acquisition as docking occurs (FBF1, TFP104, TFP101, TFP61, TFP39, TFP59, TFP49, TFP64, BBP96, BBP86, TFP123, TFP131, TFP17, TFP72 and TFP82) (Fig. 3A). In contrast, CEP164A is an example of later recruitment to mature basal body transition fibres after probasal body assembly; in such cases. a second signal was not detectable until there were four SAS-6 foci (Fig. 3B, S-phase cells). We found that 13 TFPs were recruited after probasal body assembly (CEP164A, TFP68, TFP52, CEP19, TFP32, TFP62, ARL3C, SPB1, CEP164B, RABL2B, CUL4B, TFP122 and CEP164C) (Fig. 3B).

Finally, CEP90 and OFD1 showed a different localisation pattern to the other TFPs. They were located on the mature and probasal body in all G1-phase cells (Fig. 3C, G1-phase cells). In S-phase cells with four SAS-6 foci, OFD1 and CEP90 were observed on the mature basal body (Fig. 3C, S-phase cells, middle panel) and probasal bodies (Fig. 3C, S-phase cells, lower panel). Therefore, OFD1 and CEP90 are the earliest recruited TFPs, as they are recruited to the new probasal bodies soon after their assembly. Taken together, the data are consistent with transition fibres having a hierarchical recruitment process of TFPs for docking and initiation of ciliogenesis.

### Differential localisation patterns of TFP122, RABL2B, TFP104, TFP39 and TFP72 reveal differences between flagella of different ages

An important advantage of working with *T. brucei* cells is the ability to determine the precise ‘age’ of the two flagella. During the cell cycle, the old flagellum remains assembled, and a new flagellum grows alongside in a consistent relative position (Fig. 1A). In previous work, we have shown that CEP164C is only recruited to the transition fibres of the old flagellum. Thus, recruitment only occurs in the third cell cycle after the mature basal body originally assembles as a probasal body. Furthermore, functional studies have revealed CEP164C to be essential for regulation of flagellum length, where it is proposed to be part of a locking mechanism to prevent further growth of the old flagellum whilst the new flagellum assembles during the cell cycle (Bertiaux et al., 2018; Bertiaux and Bastin, 2020; Atkins et al., 2021). Analysis of images of all 30 tagged TFPs revealed that whereas most appeared to have an equal intensity at the base of old and new flagella, TFP122, RABL2B, TFP104, TFP39 and TFP72 exhibited intensity differences between the two flagella.

**Fig. 4.**
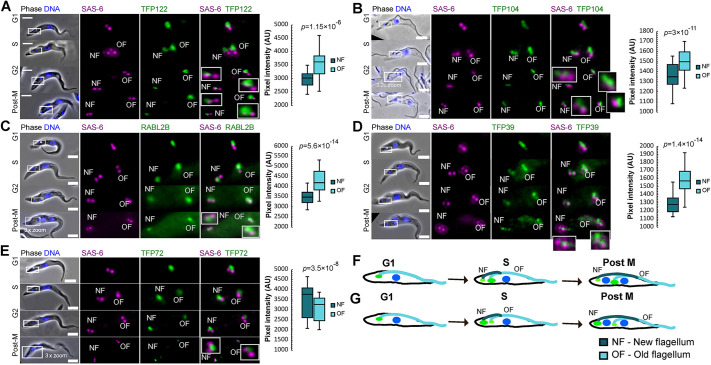
**Differential localisation of TFPs at the new and old flagellum.** (A–E) Live-cell images showing (left) the localisation throughout the cell cycle and (right) quantification of signal intensity at the old flagellum (OF) and new flagellum (NF) of TFP122 (A), TFP104 (B), RABL2B (C), TFP39 (D) and TFP72 (E). TFPs were endogenously tagged with mNG (green) and SAS-6 was endogenously tagged with mScarlet (magenta). DNA was stained using Hoechst 33342 (blue). Cell cycle stages are approximately indicated as G1 phase (two basal bodies, one kinetoplast, one nucleus), S phase (four basal bodies, one kinetoplast, one nucleus), G2 phase (four basal bodies, two kinetoplasts, one nucleus) and post-mitotic (post-M; four basal bodies, two kinetoplasts, two nuclei). Boxes in the merged phase-contrast and fluorescence images indicate the basal body region, which is shown at higher magnification in the fluorescence images on the right (5× zoom unless indicated otherwise in the figure; for some images, basal bodies are further magnified in inset images). Scale bars: 5 µm. Box plots show fluorescence signal intensity of the indicated TFP at the NF and OF, with the mean (line), interquartile range (box) and range (whiskers) marked. *n*=100 cells. *P*-values shown were calculated using a Mann–Whitney U test. (F) Cartoon illustrating increased TFP signal intensity (green) at the transition fibres at the base of the old flagellum through the trypanosome cell cycle. (G) Cartoon illustrating increased TFP signal intensity (green) at the transition fibres at the base of the new flagellum through the trypanosome cell cycle. In F and G, nuclei are shown in blue.

In cells expressing mNG-tagged RABL2B, TFP39, TFP104 or TFP122, there was a significantly higher mNG signal intensity on the transition fibres of the assembled old flagellum relative to that on the transition fibres of the assembling new flagellum in dividing cells (Fig. 4A–D, F), similar to the distribution observed for CEP164C. This differential level of expression remained throughout the cell cycle. We can conclude that these proteins are upregulated following cytokinesis when the flagellum has reached the correct length. Finally, TFP72 was identified as having a significantly higher signal intensity on the transition fibres of the assembling new flagellum throughout the cell cycle (Fig. 4E,G), suggesting that the protein is upregulated in the new flagellum during assembly and is then downregulated once assembly is complete at the end of the cell cycle.

### A functional screen reveals essential roles for TFPs in flagellum assembly and length regulation

In order to establish whether TFPs are essential for flagellum assembly or flagellum length regulation, a total of 17 TFPs were selected based on their bioinformatic and/or expression profiles for functional studies using the well characterised stable inducible RNAi-mediated knockdown system in trypanosomes (Bellofatto and Palenchar, 2008). RNAi constructs for the 17 TFPs were integrated into a cell line expressing the target TFP endogenously tagged with mNG, for use as a reporter of knockdown, and CEP164A endogenously tagged with mScarlet to establish whether knockdown of the TFP perturbed flagellum growth and/or CEP164A localisation. Stable cell lines were generated, and then individual RNAi cell lines were induced with doxycycline and compared with the uninduced cell population. Inspection of mNG fluorescence by microscopy confirmed knockdown of target TFPs from 24 h post-induction of RNAi. To assess defects in flagellum assembly and length regulation, cells were labelled with monoclonal antibodies mAb25 (Dacheux et al., 2012), which labels the axoneme of the flagellum, and BBA4 (Woodward et al., 1995), which labels basal bodies. Length measurements were taken in cells with one flagellum (G1-phase cells) before the start of the cell cycle and in post-mitotic cells at the end of the cell cycle. Post-mitotic cells contain both the existing old flagellum and new flagellum, which grows throughout the cell cycle (Sherwin and Gull, 1989) (Table S2).

**Fig. 5.**
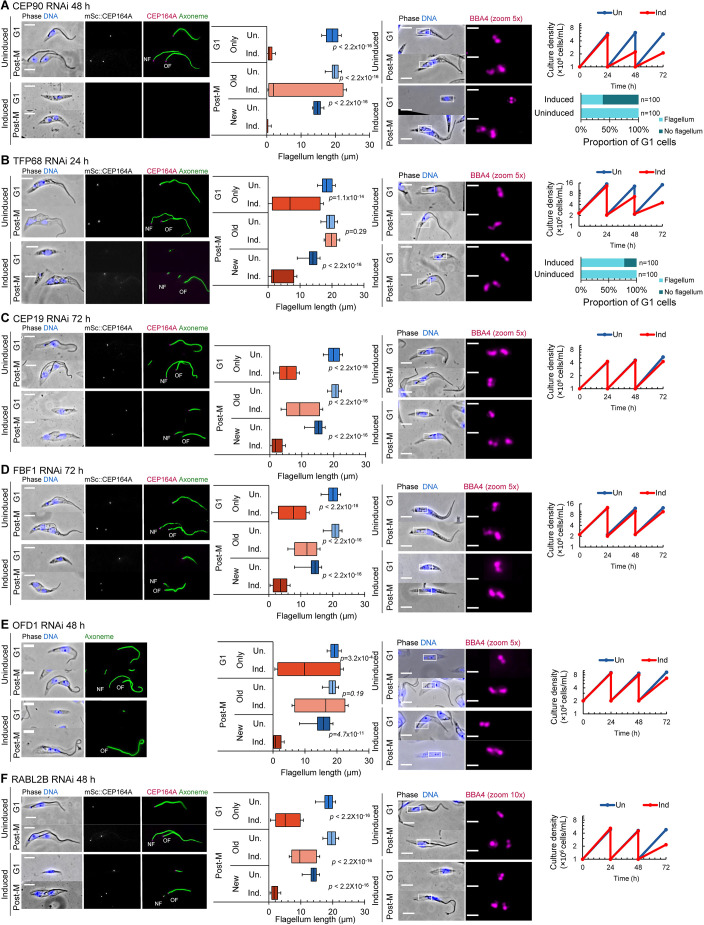
**Functional analysis of TFPs essential for new flagellum assembly.** (A–F) RNAi-mediated knockdown analysis of CEP90 (A), TFP68 (B), CEP19 (C), FBF1 (D), OFD1 (E) and RABL2B (F). Left panels: representative images of methanol-fixed cytoskeletons from G1-phase and post-mitotic (Post-M) uninduced cells and cells after induction of RNAi for the indicated times, alongside measurements of the axoneme length in G1-phase cells and the new and old flagella of post-mitotic cells for uninduced (Un.) cells and cells following induction of RNAi (Ind.). In the merged phase-contrast and fluorescence images, DNA is stained using Hoechst 33342 (blue). CEP164A was endogenously tagged with mScarlet (mSc::CEP164A; grey in single channel image, magenta in merged image) in each RNAi cell line (except OFD1 RNAi), and axonemes were labelled using mAb25 (green). NF, new flagellum; OF, old flagellum. Flagellum length box plots show the mean (line), interquartile range (box) and the 5th and 95th percentiles (whiskers). *n*=100. *P*-values were calculated using a Mann–Whitney U test. Right panels: representative images showing immunolabelled basal bodies (using BBA4, magenta) in methanol-fixed cytoskeletons from G1-phase and post-mitotic uninduced cells and cells after induction of RNAi for the indicated times, alongside growth analysis of cells depleted by RNAi (Ind, orange) and uninduced cells (Un, blue) up to 72 h, as well as the percentage of G1-phase cells with no visible flagellum for CEP90 RNAi cells (A) and TFP68 RNAi cells (B). In the merged phase-contrast and fluorescence images, DNA is stained using Hoechst 33342 (blue), and boxes indicate the basal body region shown in the magnified fluorescence images. Images are representative of three repeats. Growth analysis plots show the results of a single experiment. Scale bars: 5 µm.

Flagellum measurements revealed that six TFPs are essential for new flagellum assembly (Fig. 5A–F). In CEP90-depleted cells, new flagellum growth was severely perturbed and cell growth was reduced (Fig. 5A, left panel). At 48 h post-induction, 85% of post-mitotic CEP90 RNAi cells contained the existing old flagellum but had a new flagellum that was less than 5 μm in length. Furthermore, 61% of G1-phase CEP90 RNAi cells had no visible flagellum (Fig. 5A, right panel). In TFP68-depleted cells, new flagellum growth was also perturbed, with very short new flagella by the end of the cell cycle (Fig. 5B) and a wide variation in flagellum length. RNAi-mediated knockdown by of CEP19 (Fig. 5C), FBF1 (Fig. 5D), OFD1 (Fig. 5E) or RABL2B (Fig. 5F) led to cells with very short new flagella, but all cells contained a flagellum, even if very short. In order to assess whether transition fibres were present, at least by the presence of CEP164, we used cell lines expressing mScarlet-tagged endogenous CEP164A. CEP164A failed to localise to the transition fibres of basal bodies with no visible flagellum in both CEP90-depleted cells and TFP68-depleted cells, indicating either that docking was likely prevented by the lack of transition fibres (Fig. 5A,B) or that transition fibres were present, but CEP164A failed to localise in the absence of CEP90 or TFP68.

Basal bodies must dock to the flagellar pocket membrane for flagellum assembly, and this docking is mediated by transition fibres. In trypanosomes, basal bodies assemble within 100 nm of the flagellar pocket membrane (Fig. S3G), which makes it very difficult to assess whether there is a docking issue. Instead, we investigated basal body assembly in cell lines where new flagellum assembly was perturbed (following RNAi-mediated depletion of CEP90, TFP68, CEP19, FBF1, OFD1 or RABL2B) using the anti-basal body antibody BBA4, which labels both the mature basal body and probasal body in all cells throughout the cell cycle. Knockdown of CEP90 or TFP68 led to the formation of cells with no visible flagellum (as described above) that were often multi-nucleated and contained multiple basal body signals (observed using BBA4) (Fig. 5A,B). None of these BBA4 foci contained CEP164A. This indicates that basal bodies might be attempting to form but fail to localise and acquire transition fibres. In summary, our data show that six TFPs are essential for new flagellum assembly, but not for basal body biogenesis. Of these, CEP90 and TFP68 exhibited evidence for defective transition fibre assembly.

**Fig. 6.**
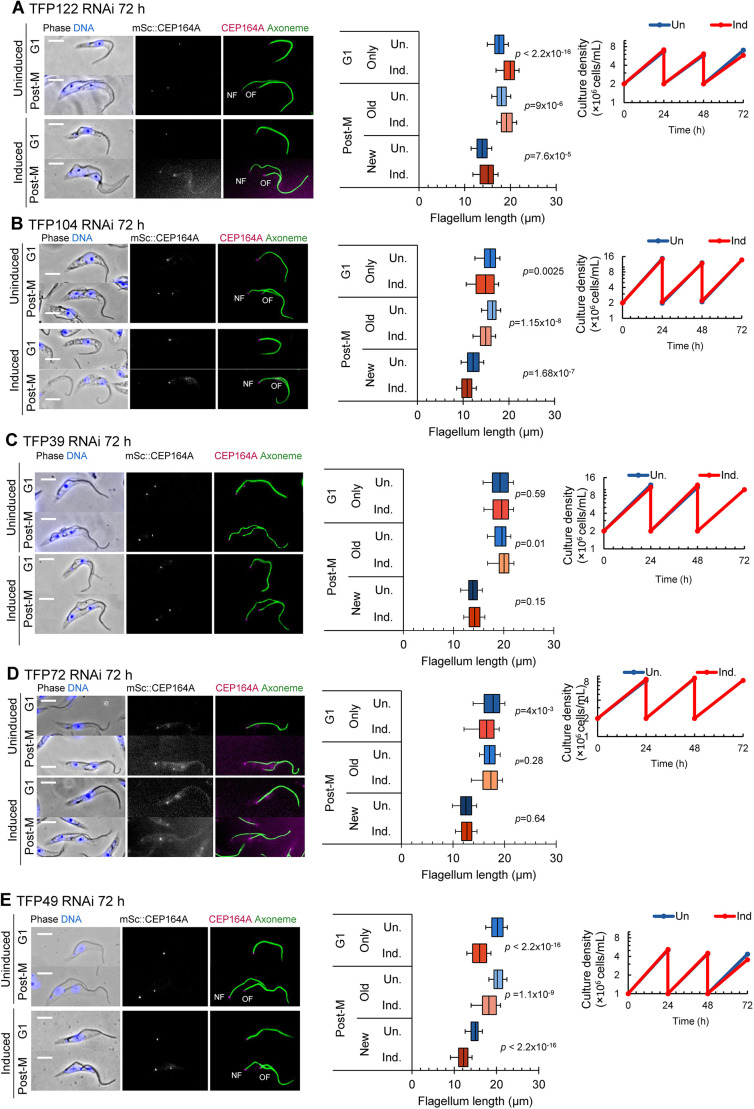
**Functional analysis of TFPs involved in flagellum length regulation.** (A–E) RNAi-mediated knockdown analysis of TFP122 (A), TFP104 (B), TFP39 (C), TFP72 (D) and TFP49 (E). Left: representative images of methanol-fixed cytoskeletons from G1-phase and post-mitotic (Post-M) uninduced cells and cells after induction of RNAi for 72 h. In the merged phase-contrast and fluorescence images, DNA is stained using Hoechst 33342 (blue). CEP164A was endogenously tagged with mScarlet (mSc::CEP164A; grey in single channel image, magenta in merged image), and axonemes were labelled using mAb25 (green). NF, new flagellum; OF, old flagellum. Scale bars: 5 µm. Middle: measurements of the axoneme in G1-phase cells and the new and old flagella of post-mitotic cells for uninduced (Un) cells and cells following induction of RNAi (Ind). Box plots show the mean (line), interquartile range (box) and the 5th and 95th percentiles (whiskers). *n*=100 cells. *P*-values were calculated using a Mann–Whitney U test. Right: Growth analysis of cells depleted by RNAi (Ind, orange) and uninduced cells (Un, blue) up to 72 h. Plots show the results of a single experiment.

Next, we asked whether RNAi-mediated knockdown of TFP122, TFP104 or TFP39, which exhibited differential localisation patterns between old and new flagella (Fig. 4), led to defective growth of new or old flagella, which might suggest a role in regulation of flagellum growth. Overall, cell growth rate was unaffected and CEP164A was retained following RNAi induction; however, measurements of flagellum length did reveal difficulties in reaching the correct flagellum length. Following knockdown of TFP122, both the single flagellum of G1-phase cells and the new and old flagella of post-mitotic cells were significantly longer compared to those of the uninduced population (Fig. 6A). After TFP104 knockdown (Fig. 6B), the old and new flagella were significantly shorter compared to those of the uninduced population. No cell growth or flagellum length defects were observed after RNAi-mediated knockdown of TFP39 (Fig. 6C). Knockdown of TFP72 (Fig. 6D) also did not reveal any flagellum length defects, despite this protein being upregulated in the transition fibres of the new flagellum of dividing cells. In summary TFPs with differential localisation patterns between new and old flagella were unable to reach the correct flagellum length.

Despite TFP49 having equal intensity on old and new flagella, knockdown of this protein also resulted in a significant reduction in flagellum length; however, the cell growth rate was still normal (Fig. 6E). Finally, depletion of five out of the six remaining TFPs chosen for the RNAi-mediated knockdown screen (TFP123, TFP17, TFP52, TFP62, SPB1) did not cause a growth defect or flagellum length defect (Fig. S4A–E). SPB1 has previously been shown to localise to the poles of the mitotic spindle during mitosis (Zhou et al., 2018), but here we found that it also localises to the transition fibres during the rest of the cell cycle, highlighting an intriguing link between two microtubule-organising centres (Figs S1, S4E). RNAi-mediated knockdown of CUL4B (Fig. S4F) caused a severe growth defect; however, flagellum assembly was not affected by CUL4B knockdown and there were no measurable differences in flagellum length between CUL4B-depleted and uninduced cells. These data indicate that CUL4B plays a role in cell cycle progression but might not be involved in flagellum formation or maintenance. Taken together, our functional analysis has identified TFPs essential for ciliogenesis and revealed further TFPs that could be important in maintaining correct flagellum length.

## DISCUSSION

### Transition fibres are complex structures

Two TFPs have previously been experimentally confirmed in trypanosomes: retinitis pigmentosa-2 (Stephan et al., 2007) and TFK1, which localises to the matrix between the transition fibre blades (Ramanantsalama et al., 2022). Our screen identified and confirmed that a further 30 proteins localise to the transition fibres in trypanosomes, bringing the total number of TFPs to 32. This challenges the traditional view that transition fibres are simple structures comprising only a few proteins. This is still likely to be an underestimate, as only localisations with a clear mature basal body-only signal from the genome-wide protein map (Billington et al., 2023) and for which we were able to establish cell lines expressing both the protein of interest and SAS-6 were taken forward in this screen. Given that mammalian and trypanosome transition fibres share morphological and functional similarities, we consider it likely that the molecular composition of transition fibres in other organisms has not yet been fully determined, and we thus speculate that there are a number of unidentified transition fibre components in other ciliated eukaryotes. The diameter of the transition fibre blades in *T. brucei* observed by scanning transmission electron tomography is ∼338 nm (Trépout et al., 2018), which is similar to our analysis and those using super-resolution microscopy techniques (Tanos et al., 2013; Yang et al., 2018; Bowler et al., 2019; Kumar et al., 2021). Furthermore, our work agrees with these studies in terms of approximate localisation diameter for the human orthologues of CEP164 and FBF1, and with the findings that CEP164 has a wider localisation diameter than FBF1 (Tanos et al., 2013; Yang et al., 2018; Kumar et al., 2021).

To date, C2CD3, OFD1, CEP83, CEP89, SCLT1, CEP164, FBF1, ANKRD26, CEP19 and CEP90 have been confirmed as TFPs in mammalian cells, but not all of these are widely distributed in an evolutionary context, even within holozoan organisms (Tischer et al., 2021). Our study confirmed that six orthologues of mammalian TFPs have the same function in trypanosomes (CEP164, FBF1, CEP90, OFD1, RABL2B and CEP19). Based on the evolutionary distribution outlined in a previous bioinformatic study (Tischer et al., 2021), along with our work, we propose that OFD1, CEP164, FBF1, CEP90, CEP19 and RABL2B are highly conserved TFPs in eukaryotes. It is likely that *T. brucei* is a highly divergent example of a universal theme in transition fibre assembly, allowing us to draw some conclusions. Firstly, some *T. brucei* proteins had hints of similarity to cilia/flagella-associated proteins in other eukaryotes based only on predicted structure, suggesting orthology, but with sequence divergence beyond the point of detectability by sequence-based methods. Secondly, α-helix-rich structural proteins likely tolerate large sequence changes while retaining function, as shown for the transition zone protein basalin (Dean et al., 2019). The α-helix-rich transition zone protein basalin is necessary for axoneme central pair assembly in *T. brucei* and *Leishmania* despite having extreme sequence divergence even between these two closely related kinetoplastids – to the point that it is only possible to confidently identify the basalin orthologues due to genome synteny (Dean et al., 2019). It is likely several α-helix-rich *T. brucei* TFPs have orthologues in wider eukaryotes that are undetectable by sequence alone.

### Timing of TFP recruitment correlates with ciliogenesis function

**Fig. 7.**
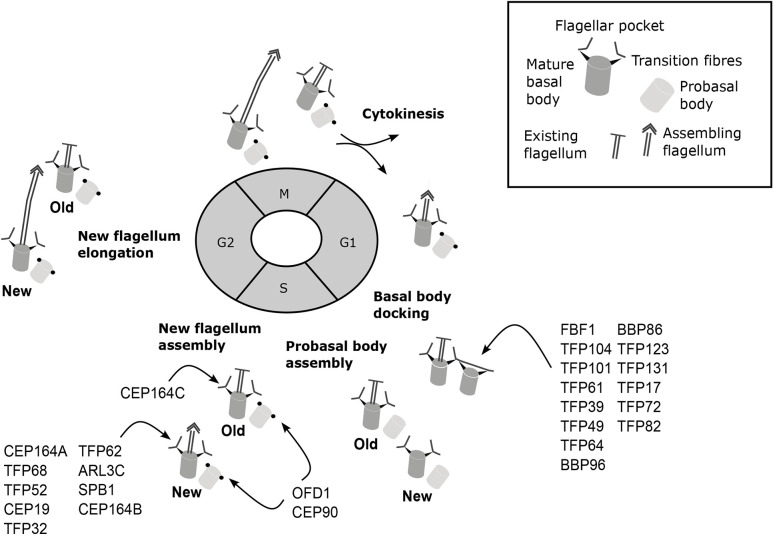
**Timing of transition fibre assembly during the *T. brucei* cell cycle*.*** Cells in G1 phase have a single flagellum and single mature basal body (dark grey) and probasal body (light grey). G1 phase: arrival of early TFPs and basal body docking to the flagellar pocket membrane. G1-S transition: probasal body assembly begins, coinciding with arrival of late TFPs. S phase: OFD1 and CEP90 are recruited to probasal bodies, and CEP164C is recruited to mature basal bodies of the existing old flagellum. G2 phase: new flagellum elongation. Cytokinesis: formation of two daughter cells, each with a single flagellum and single basal body pair.

Trypanosomes have a centriole and basal body cell cycle similar to that of mammalian cells, with probasal body duplication also occurring at the G1-S transition. A major difference is in the timing of basal body docking to form a flagellum, which occurs during S phase immediately before probasal body assembly in trypanosomes (Gluenz et al., 2011; Wheeler et al., 2019), unlike mammalian cells, where basal body docking and ciliogenesis occurs following cytokinesis. Our work shows that, with the exception of OFD1 and CEP90, TFPs arrive at the newly maturing basal body either immediately before docking or immediately after docking as a new flagellum begins assembly. We identified 15 TFPs arriving at the newly maturing basal body immediately prior to its docking to the flagellar pocket, suggesting roles in membrane docking or initiation of flagellum assembly. Of these 15 proteins, only FBF1 exhibited a ciliogenesis defect following knockdown by RNAi, suggesting a high level of redundancy. In contrast, knockdown of three of the 13 TFPs (TFP68, CEP19 and RABL2B) arriving at the maturing basal body immediately after docking resulted in defective ciliogenesis. Both OFD1 and CEP90 were found to arrive on the probasal bodies in the previous cell cycle during S phase, allowing us to conclude that OFD1 and CEP90 are the first TFPs to be recruited to the probasal body prior to maturation in the next cell cycle. Furthermore, depletion of CEP90 and OFD1 by RNAi resulted in ciliogenesis assembly defects, suggesting roles in transition fibre formation prior to ciliogenesis or initiation of ciliogenesis (Fig. 7). Indeed, recent studies have demonstrated that CEP90 and OFD1 are involved in building distal appendages in mammals (Kumar et al., 2021). In mammalian cells, TFPs are recruited to the newly maturing basal body at different points during the cell cycle, with the appearance of C2CD3 and OFD1 in G2 phase, which is followed in early mitosis by CEP83, CEP89 and SCLT1. Finally, FBF1, CEP164 and ANKRD26 appear in late mitosis, with docking occurring following cytokinesis. We can conclude that differences in the timing of TFP recruitment to mature basal bodies in different organisms is likely linked to the differences in the timing of ciliogenesis.

### Transition fibres and ciliogenesis

Several studies have shown that TFPs have essential roles in ciliogenesis, and in our work we show that six proteins (CEP90, TFP68, FBF1, CEP19, OFD1 and RABL2B) are critical for flagellum assembly. Of these, knockdown by RNAi of CEP90 and TFP68 resulted in a loss of CEP164A from the TFs, suggesting that they are also required for correct transition fibre formation. This set of essential proteins include known ciliopathy proteins that have already been implicated in biogenesis of cilia (Focşa et al., 2021). CEP90 is essential for cilium assembly in mammalian cells and forms part of a ciliopathy complex with OFD1, which is located at the distal end of mother centrioles in mammals (Kumar et al., 2021). In *C. elegans* and mammals, FBF1 regulates entry of IFT-A complexes at the ciliary base (Wei et al., 2015), whereas RABL2B and CEP19 have been shown in mammals to interact with the IFT-B complex and function in cilium assembly (Nishijima et al., 2017). Finally, in our study we show that TFP68 is also required for flagellum formation in trypanosomes and is conserved in other disease-causing kinetoplastid parasites, such as *Leishmania* spp. and *Trypanosoma cruzi*; thus, TFP68 might represent a kinetoplastid-specific flagellum assembly protein.

In our study we found that basal body duplication was unaffected and basal bodies remained in pairs and located close to the expected site of new flagellum formation following knockdown of TFPs. We can therefore conclude that TFPs are not required for basal body duplication, but we cannot be certain at what step of docking they are necessary. Due to the close proximity of the mature basal body and probasal body to the flagellar membrane, it is very difficult to assess basal body docking issues. However, knockdown by RNAi of CEP90 and TFP68 did lead to formation of cells with no visible flagellum and a lack of CEP164A. This lack of CEP164A indicates a defect with formation of the transition fibres and, as transition fibres are required for docking, suggests that a docking defect may be occurring. Further work would be required to confirm this.

### Regulating old flagellum length during new flagellum assembly

A fundamental question in cilia biology is how organisms can faithfully coordinate the assembly of a flagellum to a specific length. In organisms such as *Chlamydomonas reinhardtii,* two flagella are assembled synchronously, where there is equal access to flagellum assembly proteins such as tubulin (Marshall and Rosenbaum, 2001). However, other ciliated or flagellated organisms do not assemble their flagella synchronously, but instead assemble a new flagellum while maintaining the length of an existing flagellum (Bertiaux and Bastin, 2020).

Recent studies have implicated TFPs in flagellum length control. CEP164C localises exclusively to the transition fibres of the old flagellum and functions as part of the old flagellum length locking mechanism in trypanosomes (Atkins et al., 2021). Although our screen did not find another TFP where the signal was completely absent on the new flagellum, we did identify five proteins (RABL2B, TFP104, TFP39, TFP122 and TFP72) with a greater abundance at the base of either the old flagellum or new flagellum throughout the cell cycle, suggesting that they might be part of this mechanism. Following RNAi-mediated knockdown of TFP122, the same phenotype was observed as for CEP164C knockdown by RNAi, with cells assembling longer flagella (Atkins et al., 2021). TFP122 contains a kinesin motor head domain, and kinesin-family proteins have previously been implicated in flagellar length control in trypanosomes (Chan and Ersfeld, 2010). Further studies will be necessary to understand the mechanistic role of TFPs in flagellum length regulation in more detail and how, in general, the transition fibres ensure fidelity of flagellum length.

We conclude that transition fibres are complex and dynamic structures comprising a set of evolutionarily conserved components required for assembly and length regulation of eukaryotic cilia and flagella. Our work indicates there may be many additional components to be identified in other ciliated eukaryotes.

## MATERIALS AND METHODS

### Cell culture

SmOxP9 procyclic *T. brucei* derived from TREU 927, expressing T7 RNA polymerase and tetracycline repressor (Poon et al., 2012), were cultured at 28°C in SDM-79 medium supplemented with 10% v/v heat-inactivated fetal calf serum (Sigma-Aldrich) (Brun and Schönenberger, 1979). For growth curves, cells were counted every 24 h using a Beckman Coulter V1 cell counter and split to 2×10^6^ cells/ml.

### Endogenously tagged cell line generation

N-terminal tagging primers for construct generation by PCR from PCR-only tagging plasmids (pPOT) were designed using http://www.leishgedit.net/Home.html. Constructs for the marker cell line TbSAS-6:mScarlet (Tb927.9.10550) and TbCEP164A:mScarlet (Tb927.5.2440) were generated using the plasmid pPOTv7 (Dean et al., 2015). The pPOTv6 plasmid with 3×Ty–mNG–3×Ty (Dean et al., 2015) was used to tag each putative TFP. Each putative TFP candidate construct was transfected into the TbSAS-6:mScarlet and/or TbCEP164A:mScarlet cell lines for colocalisation. For transfection, the PCR products were combined with 1×10^7^ cells/ml and transfected using the Amaxa Nucleofactor II system. Transfectants were selected with 20 μg/ml blasticidin or 5 µg/ml G418.

### Stable inducible RNAi cell lines

Primers for RNAi were designed using RNAit (https://dag.compbio.dundee.ac.uk/RNAit/; Redmond et al., 2003). All constructs for RNAi were generated using the pQuadra stem-loop vector (Inoue et al., 2005). The parental SmOxP9 cell line was transfected with a pQuadra stem-loop plasmid containing the gene of interest and selected with 10 µg/ml phleomycin (Melford Laboratories). Transfection of SmOxP9 cells was performed using a standard electroporation protocol (McCulloch et al., 2004).

### Light microscopy

Light microscopy was carried out using a Zeiss Axio Imager Z2 widefield microscope with an ORCA-flash 4.0 Hamamatsu camera (Okerkochen, Germany) and a 100× NA 1.46 phase contrast oil immersion objective. All images were acquired using Zeiss Zen Blue software. For live-cell imaging, 1×10^7^ cells were harvested from culture in log phase (5×10^6^ to 1×10^7^ cells/ml) by centrifugation for 4 min at 800 ***g***. The pellet of cells was washed once in Voorheis modified PBS (vPBS; 137 mM NaCl, 3 mM KCl, 16 mM Na_2_HPO_4_, 3 mM KH_2_PO_4_ and 46 mM sucrose), resuspended in 1 μg/ml Hoechst 33342 in double-distilled H_2_O (ddH_2_O) for 5 min, and washed twice more and resuspended in vPBS. 1 μl of suspension was settled on an 8-well diagnostic glass slide (Epredia, X2XER203B) and mounted with a #0 coverslip (Thermo Fisher Scientific, 10011913).

### Fixation methods for light microscopy imaging

Quantitative analysis of pseudo-diameter and distance from SAS-6 measurements were carried out on methanol-fixed cytoskeletons to ensure that the cells were flat to the slide. 1×10^7^ cells were harvested in log phase (5×10^6^ to 1×10^7^ cells/ml) and centrifuged at 800 ***g*** for 4 min. Cells were washed in vPBS twice and resuspended in vPBS before settling onto slides. Cytoskeleton extract was carried out using 1% IGEPAL-NP40 (Sigma-Aldrich, I8896) in PEME buffer (100 mM PIPES-NaOH pH 6.9, 1 mM MgSO_4_, 2 mM EGTA and 0.1 mM EDTA) before incubation in methanol and being left for 30 min at −20°C. Slides were rehydrated in PBS at room temperature for 5 min and stained with 1 μg/ml Hoechst 33342 in ddH_2_O.

### Expansion microscopy

Cells were harvested and pelleted by centrifugation for 4 min at 800 ***g*** and washed twice in vPBS before being resuspended in fixation solution consisting of 4% formaldehyde and 4% acrylamide, and transferred to a 12 mm round coverslip (Sigma, P4707) coated in poly-L-lysine and placed in a 24-well plate. Cells were fixed overnight at room temperature.

For gelation, 90 µl monomer solution [19% sodium acrylate (Sigma, 408220), 10% acrylamide and 0.1% N, N′- methylenebisacrylamide (Sigma, M7256) in PBS] was combined with 5 µl of 5% N,N,N′,N′-tetramethylethylenediamine (TEMED; Sigma, T9281) and 5 µl of 5% ammonium persulfate (APS; Thermo Fisher Scientific, 17874). A 50 µl drop of monomer solution was added to a strip of parafilm in a humidity chamber on ice. Coverslips (with cells facing down) were placed on top of the drop and incubated for 5 min, then transferred to 37°C and incubated for a further 30 min. Gels were detached using a few drops of ultrapure water.

Specimens were transferred to denaturation buffer [50 mM Tris(hydroxymethyl)aminomethane hydrochloride (Sigma, 77-86-1), 200 mM sodium chloride (Sigma, S9888) and 200 mM sodium dodecyl sulphate (Fisher Scientific, 151-21-3), pH 9] in a 6-well plate. Gels were transferred to a microcentrifuge tube containing 1 ml denaturation buffer and denatured at 95°C for 90 min. Denaturation was followed by 3×20 min expansions in ultrapure water in a 10 cm dish. A 10×10 mm section of gel was cut for antibody staining. Antibodies were diluted in 2% bovine serum albumin (BSA), and gels were incubated with 500 µl of primary antibody overnight, washed in ultrapure water (3×20 min), then incubated with secondary antibody overnight followed by 3×20 min washes in ultrapure water. Specimens were finally incubated with 10 µg/ml Hoechst 33342 (Thermo Fisher Scientific, 622249) in PBS for 30 min. Cells were imaged on a 35 mm glass-bottom imaging dish (Thermo Fisher Scientific, 150680) using a Zeiss 880 confocal with AIRYSCAN and a 63× planAPO objective.

### Antibodies and immunofluorescence

Slides were pre-blocked with 1% BSA in PBS for 30 min. Primary antibodies were diluted in PBS containing 1% BSA. Samples were incubated with primary antibodies in a humidity chamber for 1 h at room temperature and washed 5×5 min in PBS. Secondary antibodies were diluted in PBS containing 1% BSA, and secondary antibody incubations were performed in a humidity chamber at room temperature for 1 h. Slides were washed 5×5 min with PBS, and DNA was stained with 1 μg/ml Hoechst 33342. Primary antibodies used were: BBA4 (1:50 dilution; kind gift from Professor Keith Gull), mAb25 (1:25 dilution; kind gift from Professor Derrick Robinson) and YL1/2 (1:10 dilution; Kilmartin et al., 1982). Secondary antibodies were: fluorescein (FITC)-conjugated AffiniPure F(ab′)_2_ fragment donkey anti-mouse IgG (H+L) (1:200 dilution; Jackson ImmunoResearch, 715096151; RRID: AB_2340796) and Rhodamine (TRITC)-conjugated AffiniPure goat anti-mouse IgG+ IgM (H+L) (1:500 dilution; Jackson ImmunoResearch, 115025044; RRID: AB_2338482), which was used with the BBA4 primary antibody.

### Transmission electron microscopy

25% glutaraldehyde (TAAB) was added to a culture suspension of SmOxP9 procyclic *T. brucei* cells at ∼5×10^6^ cells/ml (total of 10 ml) to a final concentration of 2.5%. Cells were pelleted and resuspended in a primary fixative containing 2.5% glutaraldehyde, 2% paraformaldehyde (Agar Scientific) and 0.1% tannic acid (TAAB) in 0.1 M phosphate buffer, pH 7.0 (Sigma-Aldrich). Cells were fixed for 2 h at room temperature. Pellets were washed with 0.1 M phosphate buffer (pH 7.0) and postfixed in 1% osmium tetroxide (Agar Scientific) in 0.1 M phosphate buffer (pH 7.0) for 1 h at room temperature. Samples were rinsed and stained en bloc for 40 min in 2% aqueous uranyl acetate (TAAB), dehydrated in an ascending acetone series (Fisher Scientific), and embedded in hard formulation Agar 100 resin (Agar Scientific). 70-nm thin sections were cut and stained using lead citrate for 5 min, followed by three washes with MilliQ water. Images were captured using a Hitachi H-7650 transmission electron microscope. The method used was described previously (Atkins et al., 2021).

### Orthologue identification

To identify orthologues of *T. brucei* TFPs, we primarily used a reciprocal best protein BLAST search (version 2.9.0), accepting reciprocal hits with E≤10^−5^, supplemented with orthogroup detection using Orthofinder (version 2.5.4, using FastME 2.1.4; Emms and Kelly, 2015, 2019). We identified orthologues in a diverse set of eukaryotes with previously described ability to form basal bodies and distal appendages (Carvalho-Santos et al., 2011). Predicted proteome sequences were primarily from NCBI genomes: GCA_001641455.1 Mp_v4 (*Marchantia polymorpha*), GCA_023343905.1 cvul (*Chlorella vulgaris*), GCF_000002825.2 ASM282v1 (*Trichomonas vaginalis*), GCF_000004985.1 V1.0 (*Naegleria gruberi*), GCF_000004695.1 dicty_2.7 (*Dictyostelium discoideum*), GCF_000091205.1 ASM9120v1 (*Cyanidioschyzon merolae*), GCF_000002595.1 v3.0 (*Chlamydomonas reinhardtii*), GCF_000214015.3 version_140606 (*Ostreococcus tauri*), GCF_000001735.4 TAIR10.1 (*Arabidopsis thaliana*), GCF_000002865.3 V1.0 (*Monosiga brevicollis*), GCF_000002985.6 WBcel235 (*Caenorhabditis elegans*), GCF_000001215.4 Release_6_plus_ISO1_MT (*Drosophila melanogaster*), GCF_000209225.1 ASM20922v1 (*Nematostella vectensis*), GCF_000203795.1 v1.0 (*Batrachochytrium dendrobatidis*), GCF_000328475.2 Umaydis521_2.0 (*Ustilago maydis*), GCF_000149755.1 P.sojae_V3.0 (*Phytophthora sojae*), GCF_000189635.1 JCVI-TTA1-2.2 (*Tetrahymena thermophila*) and GCF_000002765.5 GCA_000002765 (*Plasmodium falciparum*). Trypanosomatid parasite predicted proteome sequences were from TriTrypDB.org version 59, part of VeuPathDB (Amos et al., 2022): TbruceiTREU927 (*Trypanosoma brucei*), TcruziDm28c2014 (*Trypanosoma cruzi*) and LmexicanaMHOMGT2001U1103 (*Leishmania mexicana*). Human and yeast predicted proteome sequences (*Homo sapiens*, UP000005640; *Saccharomyces cerevisiae*, S288C) were from UniProt.

### Protein domains and protein structure prediction

Protein domains were identified using the *T. brucei* genome database at TriTrypDB.org, part of VeuPathDB (Amos et al., 2022). Protein structures were predicted using AlphaFold2 (Jumper et al., 2021) using a previously described custom sequence database to generate the input multiple sequence alignments (Wheeler, 2021), to provide better sequence diversity of the Discoba lineage in which *T. brucei* sits. Predicted protein secondary structure was extracted from the three-dimensional predicted structure using DSSP (Kabsch and Sander, 1983), summarising structure term ‘H’ for α-helices and ‘E’ or ‘B’ as β-sheets. Protein structures were visualised using PyMOL (https://pymol.org/). Protein structure searches against PDB were carried out using the FoldSeek server (van Kempen et al., 2024).

### Automated image analysis for measurements of distance and pseudo-diameter

An ImageJ/FIJI (https://imagej.net/software/fiji/downloads) macro was developed for automated analysis of the distance between the centres of the mNG and mScarlet signals for each tagged cell line co-expressing an mNG-tagged TFP (TFP::mNG) and mScarlet-tagged SAS-6 (SAS-6::mSc) to determine the dimensions of the tagged TFP structures. SAS-6 and tagged TFP protein signal foci in the red and green fluorescence images were first either selected manually using the ImageJ/FIJI point selection tool or identified automatically using the ‘Find Maxima’ function in ImageJ/FIJI. The precise centre points were determined using a Gaussian fit of signal in the *x* and *y* directions for each point in the green and red fluorescence images separately. TFP::mNG and SAS-6::mSc foci lie very close (within ∼500 nm), within the same cell, so the distance from each focus to the nearest SAS-6 focus, excluding distances >500 nm, was taken as the distance between these structures. To determine the dimensions of the TFP structure, the centre point of the mNG-tagged TFP structure in the green fluorescence image was identified as above. Next, signal intensity across the TFP structure was sampled in 45 straight lines spanning across the centre point, with the lines positioned at 0° (horizontal) through to 180° at 4° steps. For each line, a Gaussian distribution was fitted, and the standard deviation was taken as an effective diameter along that line. The largest effective diameter was taken as the TFP structure pseudo-diameter, excluding outliers >∼500 nm.

### Flagellum measurements and statistical analysis

Measurements of the axoneme were performed using FIJI. G1-phase cells (a single nucleus) and post-mitotic cells (two separate nuclei) were identified by Hoechst 33342 staining of the DNA. The immunolabelled axoneme (labelled with mAb25 – see above) was traced using a segmented line, and a curve was added using the ‘fit spline’ function in FIJI. Measurements were recorded and transferred to a Microsoft Excel spreadsheet. Box plots representing flagellum lengths were generated using Microsoft Excel. Statistical analysis of flagellum length (Mann–Whitney U test) was performed in R studio.

### Quantification of fluorescence microscopy signal intensity

All measurements of intensity of fluorescence signal were made in FIJI. For quantification of transition fibre signal intensity between basal bodies in dividing cells, a rectangular area of interest of a defined size was generated, and the integrated signal density was measured within the box. Measurements were made at the transition fibres and around the posterior end of the cell to measure the background signal. The background signal was subtracted from the transition fibre signal at the base of the new flagellum and old flagellum (*n*=100).

## Supplementary Material

10.1242/joces.261692_sup1 Supplementary information

Table S1. TFP general. Columns from left – Gene ID accession numbers for all TFPs taken forward. Name of each transition fibre protein determined by either orthologue names or molecular weight. Mean pseudodiameter (nm) (Figure 1E) for each TFP. Mean proximal-distal measurement (nm) (Figure 1D) for each TFP. The number of cells measured for each TFP. Co-localisation with CEP164A. The timing of recruitment to TF before or after initiation of ciliogenesis.

Table S2. RNAi. RNAi flagellum length statistical analysis, growth analysis and raw flagellum length data in μm.
