# Supplementary material for: Identification of 30 transition fibre proteins in Trypanosoma brucei reveals a complex and dynamic structure
Source: J Cell Sci. 2024 Jun 3;137(10):jcs261692. doi: 10.1242/jcs.261692 (PMC11190437; doi:10.1242/jcs.261692)
Supplement: Supplementary information [file joces-137-261692-s1.pdf]

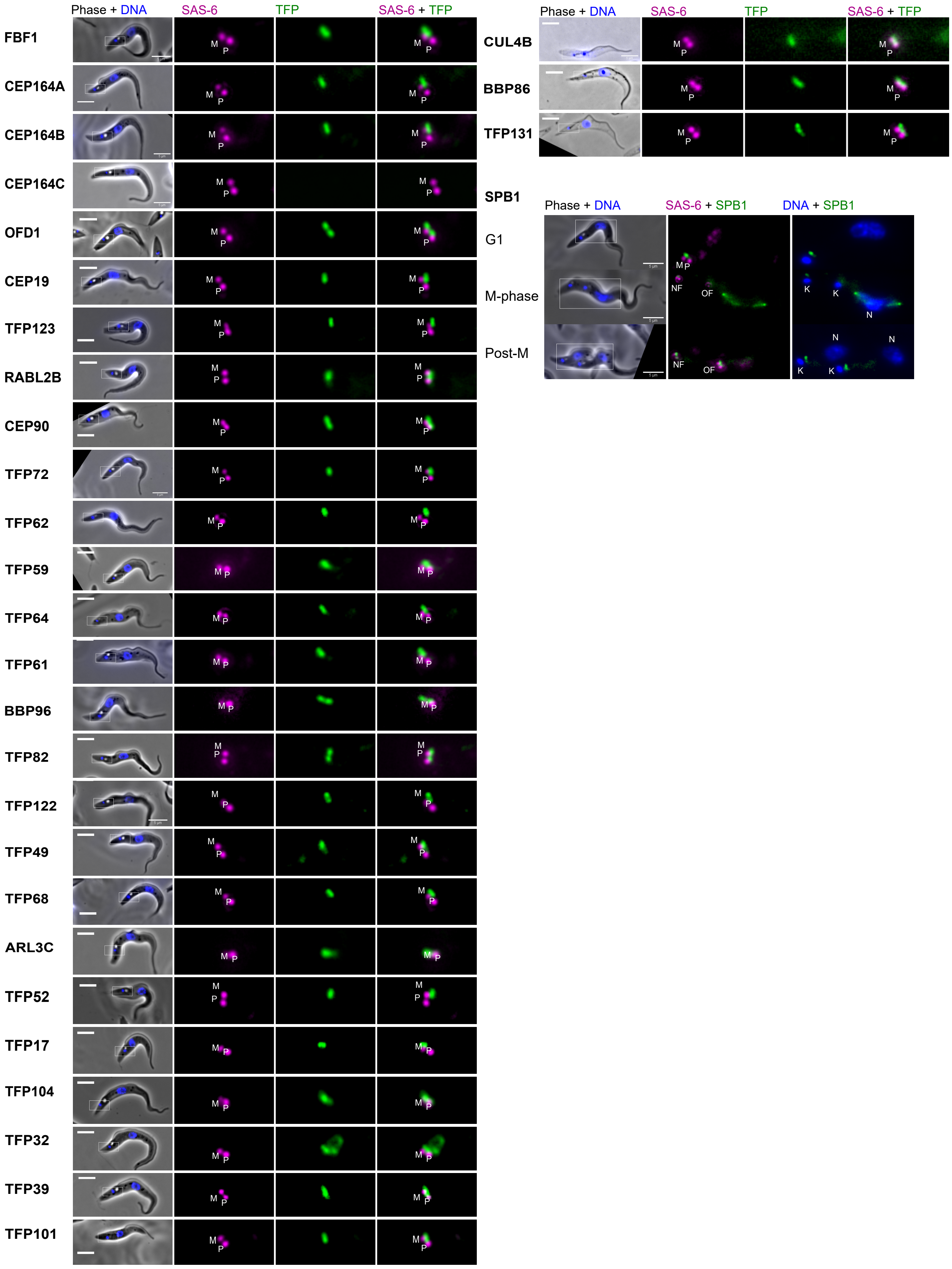

Fig. S1. Localisation of 30 endogenously tagged putative transion fibre proteins (TFPs). TFPs endogenously tagged with SAS-6:mScarlet (magenta). A G1 cell is shown for each tagged TFP. Scale bar = 5 µm. M – mature basal body, P – robasal body. Insets represents a 5x zoom. Note, CEP164C is only present on the old flagellum during the cell division cycle and shown here as absent from a G1 cells - see text. Three cell cycle stages shown for SPB1. In G1 cells the signal is only at the transition fibres, during mitosis (M-phase) signal is present on transition fibres and poles of the mitotic spindle, but does not remain postmitosis when 2 nuclei are visible.

Journal of Cell Science • Supplementary information

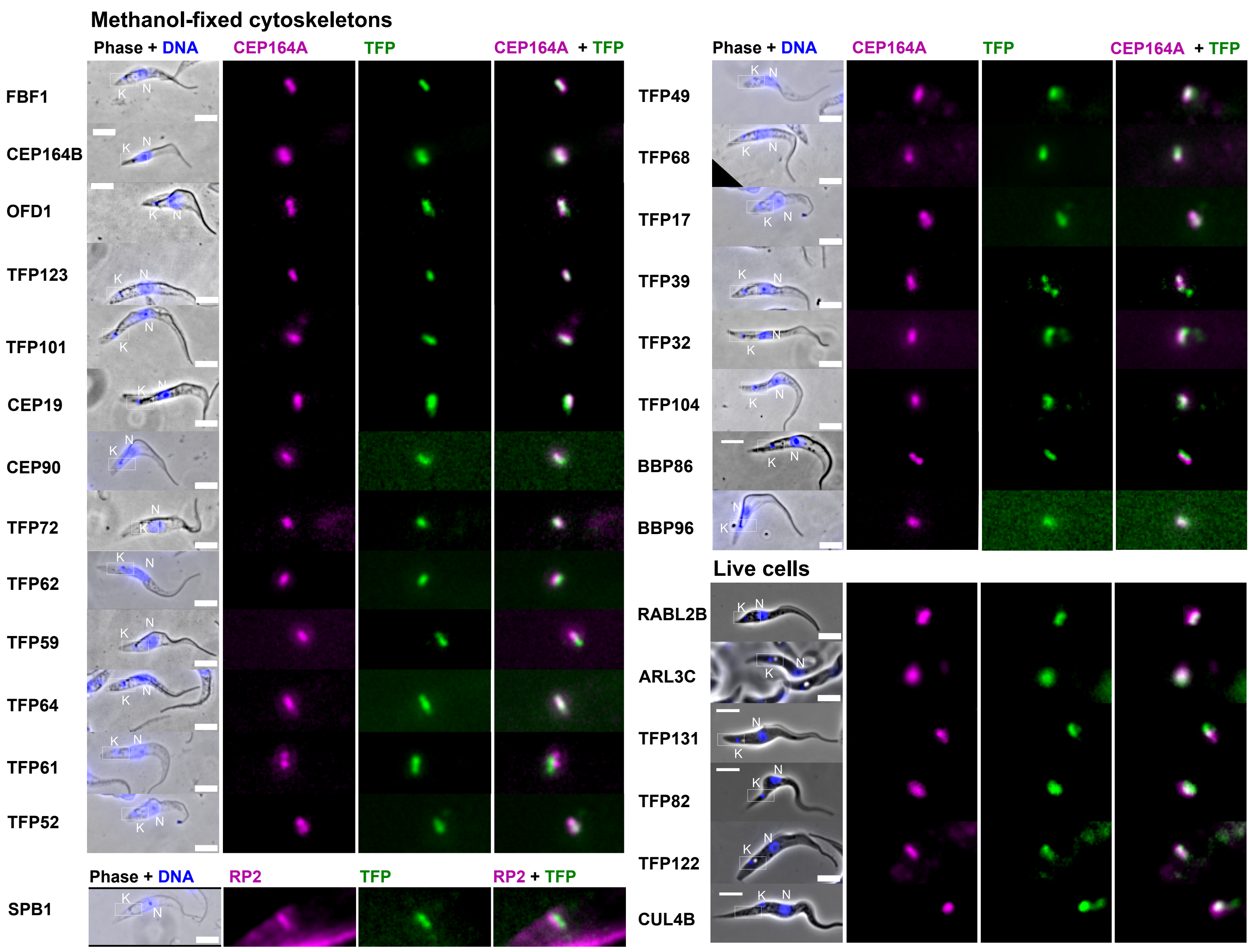

Fig. S2. Co-localisation of TFPs with CEP164a or RP-2. Cell lines endogenously tagged with CEP164A mScarlet (magenta) or immunolabelled RP2 with the YL1/2 antibody. A G1 cell is shown for each TFP. Scale bar = 5μm, K-Kinetoplast, N- Nucleus. Inset represents 5x zoom.

Journal of Cell Science • Supplementary information

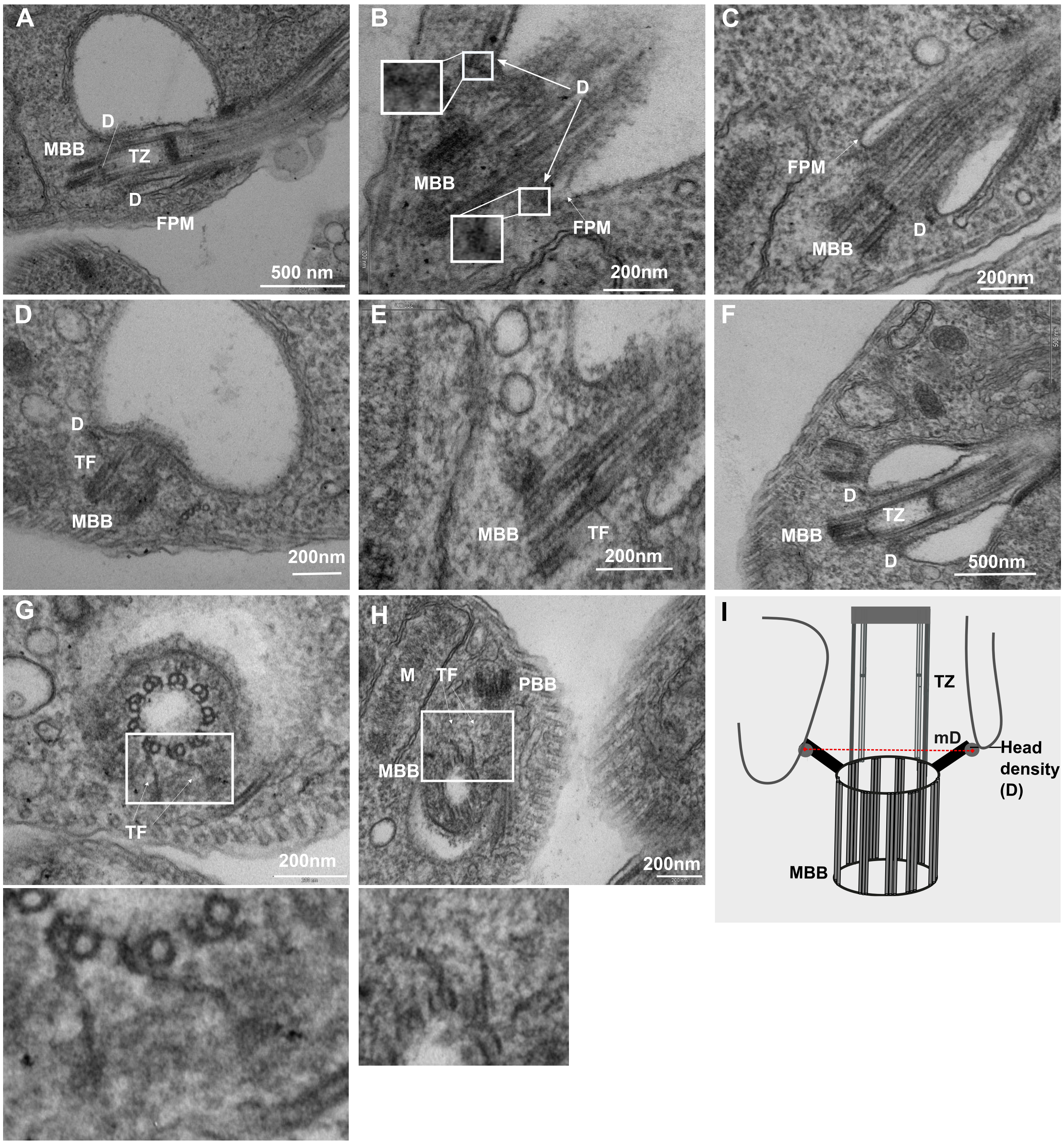

Fig. S3. Measurements of Transition Fibres by transmission electron microscopy (TEM). A,B,C,D,E,F: Longitudinal sections of organisation of flagellar pocket membrane (FPM), Mature basal body (MBB), Probasal body (PBB), transition fibre densities (D), transition fibres (TF) and transition zone (TZ). G, H: Cross section of transition fibres. I: Cartoon of transition fibre and basal body with illustration of measurement to confirm axial width of transition fibres. Inset shows a 5x zoom.

Journal of Cell Science • Supplementary information

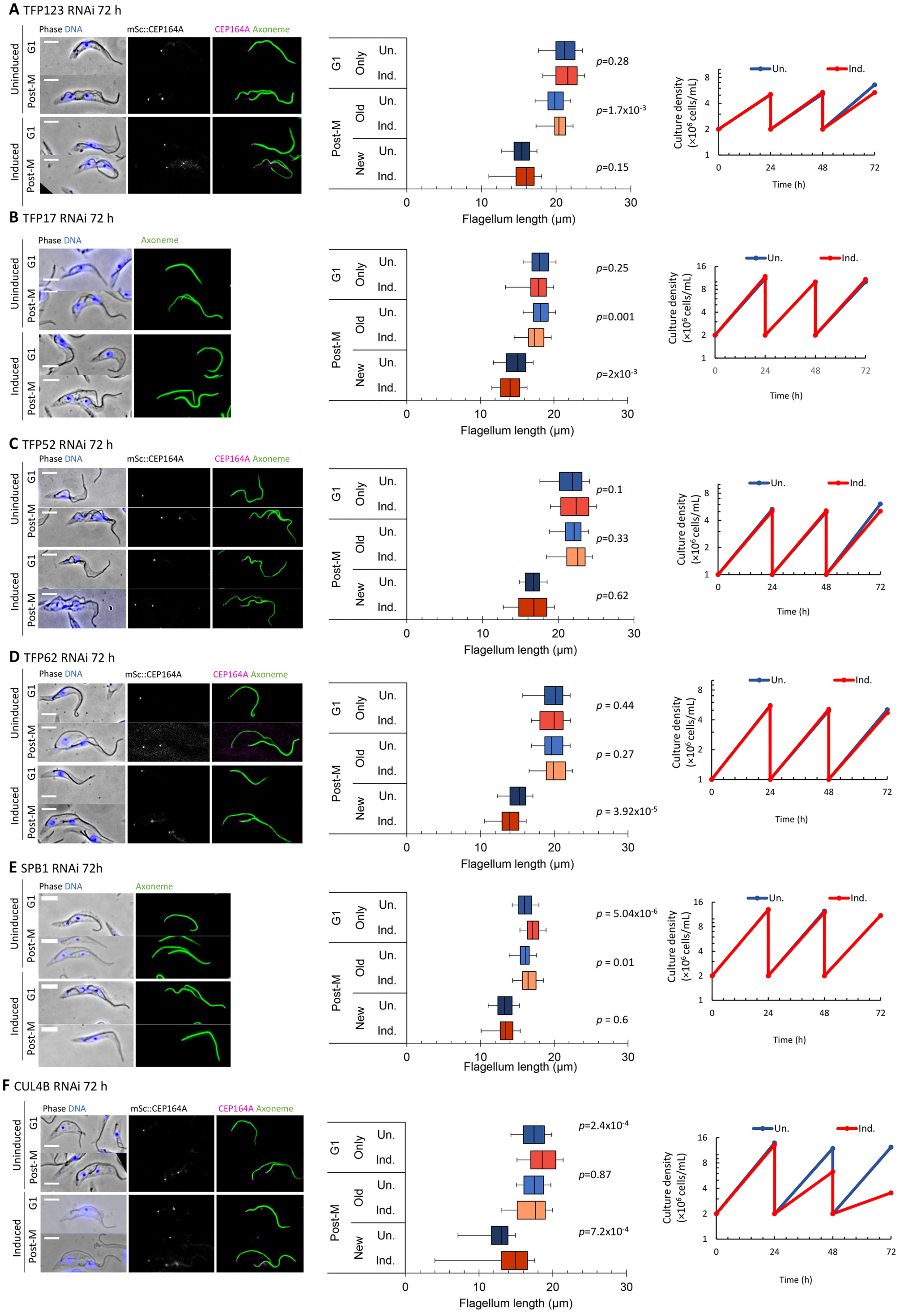

Journal of Cell Science • Supplementary information

Fig. S4. RNAi analysis of TFPs with no change in flagellum length. RNAi knockdown of TFP123 (A), TFP17 (B), TFP72 (C), TFP62 (D), SPB1 (E), CUL4B(F). Panels from left: Representative images of methanol-fixed cytoskeletons with uninduced cells and cells post induction of RNAi (induced). Endogenously tagged CEP164A in each RNAi cell line (except for TFP17 and SPB1) shown in magenta and axoneme (mAb25) in green; Measurements of the axoneme (µm) in G1 cells and the new and old flagella of post mitotic cells in uninduced (Un) and induced following induction of RNAi (Ind.) (n=100); Growth analysis of cells depleted by RNAi (red – Ind) and uninduced cells (blue – Un) up to 72 hours; Scale bar = 5µm. NF – new flagellum, OF – old flagellum.

Table S1. TFP general. Columns from left – Gene ID accession numbers for all TFPs taken forward. Name of each transition fibre protein determined by either orthologue names or molecular weight. Mean pseudo-diameter (nm) (Figure 1E) for each TFP. Mean proximal-distal measurement (nm) (Figure 1D) for each TFP. The number of cells measured for each TFP. Co-localisation with CEP164A. The timing of recruitment to TF before or after initiation of ciliogenesis.

Available for download at
https://journals.biologists.com/jcs/article-lookup/doi/10.1242/jcs.261692#supplementary-data

Table S2. RNAi. RNAi flagellum length statistical analysis, growth analysis and raw flagellum length data in µm.

Available for download at
https://journals.biologists.com/jcs/article-lookup/doi/10.1242/jcs.261692#supplementary-data
